# Evaluation of early antioxidant and anti-inflammatory effects of *Ecballium elaterium* (L.) A. rich balm in rats with imiquimod-induced psoriasis

**DOI:** 10.3389/fphar.2026.1876388

**Published:** 2026-07-13

**Authors:** Djihene Narimene Benmalek, Fatima Zohra El Kadi, Aicha Benyahia, Mahedi Hassan Tusher, Omar Kharoubi, Noria Harir, Dalia I. Hemdan, Rokayya Sami, Sarah Alharthi, Awatif Almehmadi, Abdullah M. Izmirly, Shatha Alzahrani, Luluah M. Al Masoudi, Ehssan A. Hassan, Aseel Alyahyawi, Abeer G. Almasoudi

**Affiliations:** 1 Laboratory of Molecular Microbiology Health and Proteomics, Department of Biology, Faculty of Natural Sciences and Life, Djillali Liabes University of Sidi Bel Abbes, Sidi Bel Abbes, Algeria; 2 Laboratory of Biotoxicology, Bioremediation and Phytoremediation, Biology Department, Faculty of Natural and Life Sciences, Ahmed Ben Bella University of Oran1, Oran, Algeria; 3 Department of Pharmacology, Faculty of Basic Sciences, Bangladesh University of Health Sciences (BUHS), Dhaka, Bangladesh; 4 Department of Food Science and Nutrition, College of Sciences, Taif University, Taif, Saudi Arabia; 5 Department of Chemistry, College of Science, Taif University, Taif, Saudi Arabia; 6 Research Center of Basic Sciences, Engineering and High Altitude, Taif University, Taif, Saudi Arabia; 7 Department of Clinical Nutrition, Faculty of Applied Medical Sciences, Umm AL-Qura University, Makkah, Saudi Arabia; 8 Department of Medical Laboratory Sciences, Faculty of Applied Medical Sciences, King Abdulaziz University, Jeddah, Saudi Arabia; 9 Special Infectious Agents Unit—BSL3, King Fahd Medical Research Center, King Abdulaziz University, Jeddah, Saudi Arabia; 10 Department of Clinical Laboratory Sciences, College of Applied Medical Sciences, Taif University, Taif, Saudi Arabia; 11 Department of Biology, College of Sciences, Taif University, Taif, Saudi Arabia; 12 Department of Biology, College of Science and Humanities in Al-Kharj, Prince Sattam Bin Abdulaziz University, Al-kharj, Saudi Arabia; 13 Department of Zoology, Faculty of Science, Suez Canal University, El-Sheikh Zayed, Ismailia, Egypt; 14 College of Medicine, Fahad Bin Sultan University, Tabuk, Saudi Arabia; 15 Program of Food Sciences and Nutrition, Turabah University College, Taif University, Taif, Saudi Arabia

**Keywords:** *Ecballium elaterium*, imiquimod, oxidative stress, psoriasis, TNF-α

## Abstract

Psoriasis is a persistent cutaneous disorder involving an imbalance in immune regulation, oxidative stress, and abnormal epidermal proliferation. This study evaluated the therapeutic efficacy of *Ecballium elaterium* (EE) balm compared with clobetasol propionate (0.05%) (CLO) in rats with psoriasis-like lesions induced by topical imiquimod (IMQ). A total of twenty-four adult male of the Wistar strain were randomly allocated to four groups comprising: control (healthy rats), IMQ-induced psoriasis, IMQ + EE balm, and IMQ + clobetasol. Psoriasis was induced by topical application of 5% imiquimod. Treatments were administered daily. Disease severity, histological changes, oxidative stress markers, serum tumor necrosis factor-α (TNF-α), and biochemical parameters were evaluated. EE balm markedly improved psoriatic lesions and reduced psoriasis severity scores in imiquimod-treated rats. Biochemical analyses indicated that EE balm produced fewer disturbances in hepatic and lipid parameters than clobetasol, notably by preventing alanine aminotransferase elevation (EE: 62.20 ± 3.09; CLO: 89 ± 6.08) and lowering total cholesterol (EE: 0.47 ± 0.08; CLO: 0.63 ± 0.03). However, serum triglyceride (EE: 0.61 ± 0.03) and creatinine concentrations (EE: 6.03 ± 0.23) remained elevated. Serum TNF-α levels, markedly elevated in imiquimod-treated rats, were significantly reduced following EE balm and clobetasol treatments compared with the IMQ experimental set (p < 0.001). EE balm also mitigated oxidative stress by lowering thiobarbituric acid reactive substances (TBARS) levels in serum and erythrocytes in the IMQ group. It also restored antioxidant activities, including catalase (IMQ: 3.06 ± 0.79; EE: 4.24 ± 0.73), super oxide dismutase (IMQ: 03.17 ± 1.01; EE: 18.24 ± 1.90), glutathione peroxidase (EE: 31.25 ± 4.99). Additionally, glutathione s-transferase activity was improved in serum and erythrocytes (EE: 09.96 ± 2.29), as were reduced glutathione levels (IMQ: 0.0021 ± 0.0007; EE: 0.0042 ± 0.001). In an imiquimod-induced psoriasis rat model, *Ecballium elaterium* balm reduced disease severity, oxidative stress, and inflammatory markers, with systemic biochemical changes. Despite these promising findings, this research is still in its early stages based on a single preclinical model.

## Introduction

Psoriasis is a kind of immune-mediated skin sickness manifested through reddish and scaly lesions, affecting nearly 4% of people globally, with varying prevalence rates differing according to geographic location (Lin et al., 2026; Xiong et al., 2025). Clinically, the disease may arise at any age, affecting many anatomical regions often putting a substantial burden on people (Lubwama et al., 2025). Plaque psoriasis is a more prevalent within clinical variations, with guttate psoriasis in second rank (Zhou et al., 2024).

Psychological stress can influence immune responses, leading to increased amounts of mediators of inflammation, such as interleukin-1β (IL-1β) and tumor necrosis factor-α (TNF-α) (Bakar et al., 2021; Alesci et al., 2022). In addition, infectious agents such as *Staphylococcus aureus*, recurring pharyngitis, and genital infections may also trigger psoriatic flare-ups by stimulating inflammatory cascades within the host’s immune system (Balato et al., 2023).

Histologically, mature psoriatic plaques display pronounced hyperkeratosis with thickening of the stratum corneum, parakeratosis with retained keratinocyte nuclei, acanthosis from excessive keratinocyte proliferation, elongation of dermal papillae, reduced granular layer thickness, increased vascularization, and inflammatory cell infiltration within the superficial dermis (Alzghool et al., 2026). Reactive oxygen species (ROS) come from endogenous sources including NADPH oxidase activity, mitochondrial oxidative phosphorylation, and xanthine oxidase-mediated reactions, as well as by exogenous factors such as UV radiation, infrared radiation, visible light, and environmental pollutants. These factors cause damage to keratinocytes, promoting the secretion of LL-37 and the release of intracellular DNA. Their interaction forms complexes that activate Toll-like receptor 9 (TLR9) in plasmacytoid dendritic cells (pDCs), leading to the production of interferon-α (IFN-α). This interferon in turn stimulates myeloid dendritic cells (mDCs) to secrete TNF-α, interleukin-12 and interleukin-23 (IL-12, IL-23), promoting Th1- and Th17-mediated immune responses (Kamata and Tada, 2023; Uchida et al., 2026). Collectively, these signaling pathways lead to persistent inflammatory responses and excessive keratinocyte proliferation.

Murine models have been reliably used to mimic psoriasiform skin inflammation using imiquimod (IMQ). IMQ is a compound that modulates immune responses and activates Toll-like receptors 7 and 8 (TLR7/TLR8). Topical application of IMQ is widely used to manage and to treat virus-induced dermatoses, actinic keratoses, and basal cell carcinoma (Van der Fits et al., 2009). This research aimed to evaluate antioxidant and anti-inflammatory effects of *Ecballium elaterium* (L.) A. Rich, belonging to the Cucurbitaceae family. This species is rich in bioactive metabolites, including flavonoid, phenolic, tannin, trypsin inhibitor, lipid, and cucurbitacin (B, D, E, I, and L), which may provide therapeutic benefits against imiquimod-induced psoriasis in Wistar rats (Arslan et al., 2016; Saeed et al., 2019). The plant is widely distributed worldwide particularly in North Africa and the Eastern Mediterranean region, its fruit has been utilized for medicinal purposes in the traditional Mediterranean pharmacopeia. In Anatolian folk medicine, the fruit is applied topically to relieve rhinosinusitis by reducing inflammation, while the roots are used as an analgesic and to alleviate hemorrhoidal symptoms (Hamidi et al., 2022; Anzano et al., 2024). Regarding the traditional use of *Ecballium elateruum* in the treatment of skin conditions, ethnobotanical studies conducted in Algeria and Turkey have reported its traditional use in the treatment of skin diseases, particularly eczema and psoriasis in several preparations, including powder, decoction, raw and maceration (İbiloğlu et al., 2021; Hantour et al., 2026). In addition, studies have highlighted its antioxidant, anti-inflammatory, and cicatrizing properties, suggesting its potential application in the treatment of inflammatory skin diseases (Erarslan et al., 2020).

Although *Ecballium elaterium* has traditionally been used to treat dermatological conditions, its therapeutic potential in the treatment of psoriasis has not yet been sufficiently studied. Current study evaluated the anti-inflammatory and antioxidant features of a natural formulation based on *Ecballium elaterium* in male Wistar rats with imiquimod-induced psoriasis. The therapeutic effects were evaluated using histological, biochemical and immunological analyses, as well as markers of oxidative stress, to enable a thorough assessment of the plant’s protective potential in this model.

## Materials and methods

### Topical ointments

A commercially available 5% imiquimod cream (Aldara®) was used for psoriasis induction. Each sachet contains 250 mg of formulation corresponding to 12.5 mg of imiquimod. The experimental model was established according to previously described protocols (Singh et al., 2019). Clobetasol propionate 0.05%, a highly potent topical corticosteroid of the dermocorticoid class, is available in pharmacies in 45 g tubes and was used as a reference treatment due to its established efficacy in experimental psoriasis models (Salman et al., 2024).

## Plant material

Mature fruits of *Ecballium elaterium* (L.) A. Rich. Subsp. *Ecballium elaterium* (Cucurbitaceae) were carefully collected in February 2024 from the Oued El Djemaâ region (Relizane, central Algeria), where the species naturally occurs in ruderal habitats along the margins of a seasonal watercourse. The collected material was placed in paper bags and transferred to the Laboratory of Biotechnology of Rhizobia and Plant Breeding (LBRAP), University of Oran 1 Ahmed Ben Bella.

The subspecies was identified by the herbarium curator, primarily using the taxonomic keys of Quézel and Santa (1962), together with data from the eFlora Maghreb online botanical database. A voucher specimen was deposited in the LBRAP herbarium under the accession code LBRAP-Cuc129.

The fruits were separated into seeds and external pericarp, then dried under the sunlight. The powder obtained after dehydration and ground was stored in sterile glass containers. To formulate the balm, the powder was macerated in olive oil at a ratio of 1:5 (w/v), equal to 40 g in 200 ml for 4 weeks at room temperature, with daily manual stirring. The preparation was then filtered, and beeswax was incorporated at 7% into the filtrate to obtain a semi-solid balm suitable for topical application.

## Animals

**FIGURE 1:**
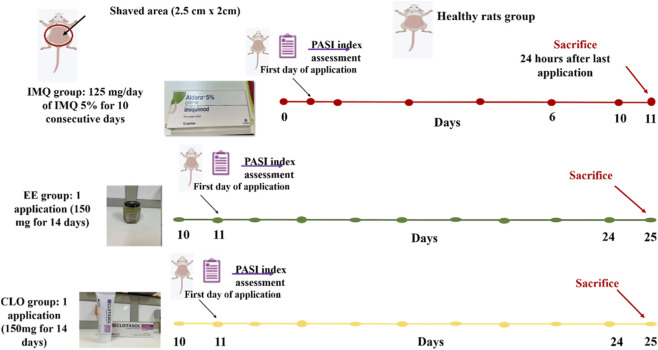
Experimental design for the induction of psoriasis in rats, and the treatments applied during the experimental trial.

Twenty-four adults male Wistar rats (aged 8 weeks, 220.05 ± 22.66 g) were obtained from the University of Oran 1. They were randomly assigned to four experimental groups (six rats per group). After shaving the back with a razor, a depilatory cream was applied to remove any remaining hair. An area of approximately 2.5 × 2 cm was prepared for topical administration. The experimental design shown in Figure 1 included:

Group I: A healthy control group.
Group II: an IMQ treatment group, receiving 125 mg of 5% imiquimod topical cream (6.25 mg of imiquimod) applied daily on the shaved surface.
Group III: *Ecballium elaterium* group (EE group), receiving a daily application of EE balm for 2 weeks following induction (150 mg).
Group IV: Clobetasol group (CLO group), receiving a daily application of clobetasol 0.05% cream for 2 weeks following induction (150 mg).

### Assessing the severity of skin inflammation (PASI)

The clinical assessment of skin irritation was conducted starting on day 0, consistent with a modified PASI score (Van der Fits et al., 2009). Erythema, desquamation, and skin thickness were assessed separately for each animal. Each criteria was rated on a scale of 0–4, ranging from 0 (no visible changes) to 4 (very marked). Intermediate scores corresponded to mild (1), moderate (2), and marked manifestations (3). The final score for each rat calculated by adding the three individual parameters, resulting in a maximum possible value of 12 reflecting increased inflammation as: PASI total=Erythema+desquamation+skin thickness (Okine et al., 2016; Okasha et al., 2018).

### Histological tests

Skin samples were immersed in 10% buffered formalin for fixation. Samples were dehydrated in ethanol solutions at increasing concentrations, clarified with toluene, and enclosed in paraffin at 56 °C. The paraffin blocks were sectioned into sections of 4 µm using a rotary microtome. The sections were placed on glass slides coated with albumin and left to dry at 37 °C. The slides were deparaffinized and rehydrated, then stained with hematoxylin and eosin (H&E) to allow histological evaluation under a light microscope (Smith et al., 2005). Epidermal thickness (µm) is defined by the distance between the surface of the epidermis and the base of the rete ridges.

### Biochemical analyses

Biochemical parameters related to lipid function, including total cholesterol (TC), high-density lipoprotein cholesterol (HDL-C), low-density lipoprotein cholesterol (LDL-C), and triglycerides (TG), were analyzed in all experimental groups. Liver function was evaluated by measuring the activities of aspartate aminotransferase (AST) and alanine aminotransferase (ALT), while kidney function was assessed through serum urea and creatinine concentrations. All assays were conducted using commercially available kits (BioMaghreb/Biosystemes, Spain), strictly following the manufacturers’ instructions. In addition, uric acid concentration was determined through an enzymatic colorimetric method based on the uricase-peroxidase reaction, and total protein content in both serum and erythrocytes was quantified using the method described by Lowry et al. (Lowry et al., 1951).

Oxidative stress was evaluated by measuring lipid peroxidation and antioxidant defense markers. Levels of thiobarbituric acid reactive substances (TBARS) were determined according to the method reported by Richard et al. to assess oxidative degradation of lipids (Richard et al., 1992). Catalase (CAT) enzyme activity was evaluated using Sinha’s colorimetric assay and reported as mmol H_2_O_2_ degraded per min per mg of protein (Sinha, 1972). Superoxide dismutase (SOD) and glutathione peroxidase (GPx) activities in the samples were evaluated according to Marklund and Marklund’s protocol (Marklund and Marklund, 1974) and Gunzler et al. (1974), respectively.

Glutathione S-transferase (GST) activity in the samples was evaluated according Habig et al. This assay is based on the conjugation of reduced glutathione with 1-chloro-2,4-dinitrobenzene (CDNB), a substrate that reacts readily with several GST isoforms (Habig et al., 1974). Reduced glutathione (GSH) were determined as described by Sedlak and Lindsay (Sedlak and Lindsay, 1968), with minor adjustments.

### Tumor necrosis factor alpha (TNF-α) immunoassay

TNF-α levels were quantified via a solid-phase sandwich enzyme-linked immunosorbent assay (ELISA) employing a pair of TNF-α-specific monoclonal antibodies and according to the manufacturer’s instructions (Invitrogen, Thermo Fisher Scientific, United States).

### Statistical analysis

All biochemical measurements for each sample were performed in triplicate. Statistical analyses were carried out with IBM SPSS (version 20.0) and GraphPad Prism (version 11) software. Biochemical and oxidative stress data were reported as mean ± standard deviation (SD), and as mean ± standard error of the mean (SEM) for PASI and TNF-α measurements (n = 6). Normality of distrubtion was assessed using the Kolmogorov-Smirnov test, and the homogeneity of variances using Levene’s test. Group comparisons were conducted using one-way ANOVA followed by Tukey’s *post hoc* test. Means accompanied by different letters indicate statistically significant differences at p* <0.05.

## Results

### Psoriasis induction

**FIGURE 2:**
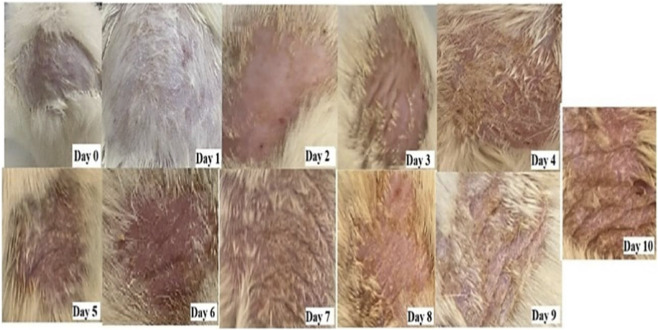
Induction and evolution of psoriatic lesions in groups treated with IMQ for 10 days.

Rats receiving 125 mg of imiquimod for ten consecutive days developed typical psoriasiform symptoms, including erythema, epidermal thickening, and scaling. Erythema became noticeable by the second day and progressively intensified through day ten. By the fourth day, both skin thickening and desquamation began to emerge, gradually worsening and reaching their maximum severity on day ten, where dome-shaped lesions became clearly visible as shown in Figure 2.

**FIGURE 3:**
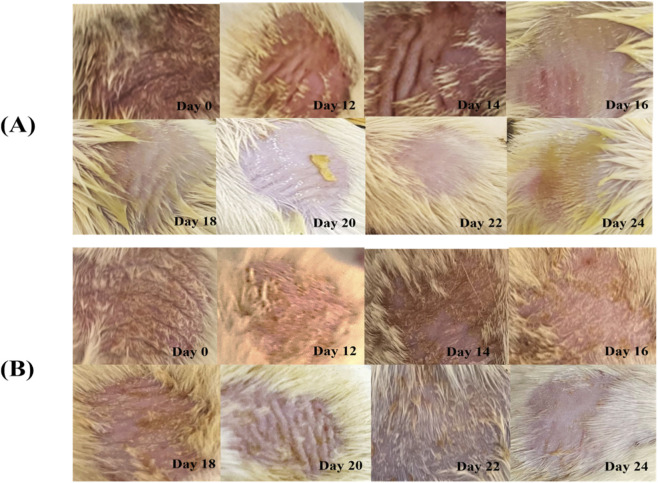
Evolution of psoriasis lesions: **(A)** EE balm treated group; **(B)** clobetasol-treated group.

Figures 3A,B present the evolution of erythema, epidermal thickening, and lesion severity across the different experimental groups. In rats receiving topical (EE) balm after psoriasis induction, a clear reduction in clinical scores was observed. Erythema decreased from day sixteen and continued to decline progressively until day twenty-four. Skin thickening and lesion severity were also visibly reduced during the treatment period.

Rats in the CLO group received daily topical application of 0.05% clobetasol (Figure 3B). A reduction in erythema and scaling was observed during the first week of treatment. Lesion severity continued to decrease until day twenty four. However, residual skin thickening remained visible at day twenty-four.

### Evolution of PASI scores

**FIGURE 4:**
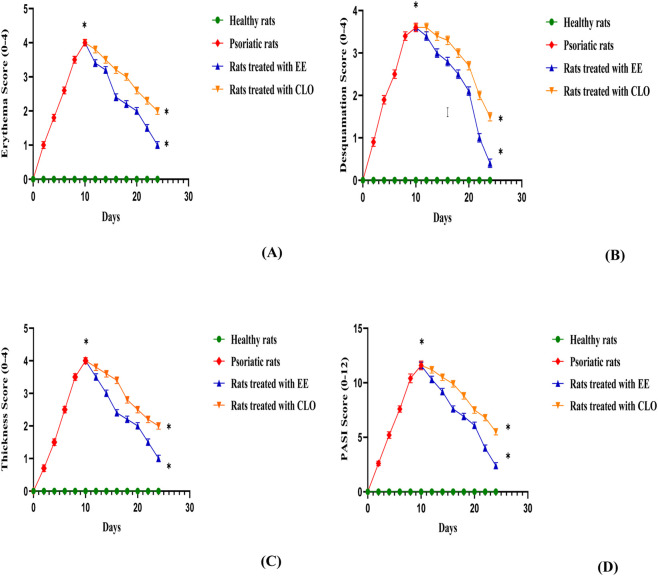
Effects of treatments on psoriasis like features: **(A)** Erythema; **(B)** Desquamation; **(C)** Thickness; **(D)** PASI inflammation score.; *p < 0.05 vs healthy group.

PASI values were assessed over experimental period of 10 days (Figure 4). Healthy animals maintained a constant score of 0.00 ± 0.00 during the study. In contrast, IMQ-treated rats showed a progressive increase, reaching 7.62 ± 0.61 on day 10.

Rats treated with EE showed a gradual decline in PASI values over the study period (6.56 ± 0.41). A reduction was also observed in the CLO group, although mean values remained higher (7.49 ± 0.40). By day 24, scores were lower in the EE group animals than in those receiving clobetasol.

**FIGURE 5:**
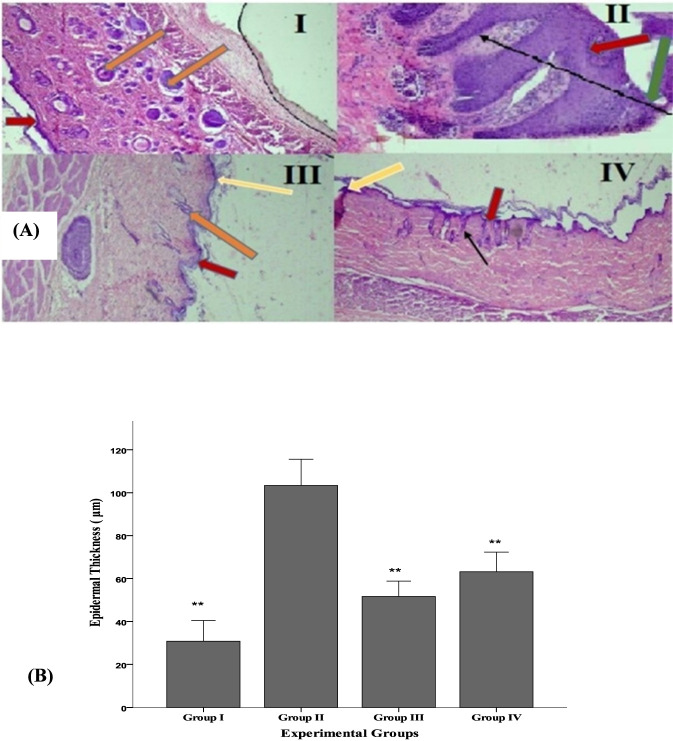
**(A)** Group I (control) normal skin structures: Epidermis (red arrow), and skin appendages (orange arrow). Group II (IMQ group) with psoriasis characteristics: marked inflammatory (red arrow), acanthotic changes (black arrow), and hyperkeratotic and parakeratotic lesions (green arrow). Group III (EE group): a thinned epidermis (red arrow), hyperkeratotic lesions (yellow arrow), and reappearing appendages (orange arrow). Group IV (CLO group): mild inflammation (red arrow), minor hyperkeratotic lesions (yellow arrow), and slight acanthotic changes (black arrow). **(B)** Epidermal Thickness (μm) across the different experimental groups. **p < 0.05 compared to the 5% IMQ group.

Histological evaluation of dorsal skin was carried out using H&E staining (Figure 5A). Among group I, the skin structure was normal, with intact keratin layers, a structured dermis, visible sebaceous glands, and clearly defined hair follicles. In contrast, group II showed marked epidermal hyperplasia with rete ridge elongation. Additional alterations were also observed, including hyperkeratosis, parakeratosis, acanthosis, and structural changes within the papillary dermis.

Skin sections from Group III revealed a marked improvement in psoriatic signs. Parakeratosis was reduced, epidermal thickness approximated normal, and hair follicles were regenerated. Reticular ridges were no longer visible, reflecting restoration of skin structure. In Group IV, histological changes were moderate. The reticular ridges were elongated, moderate hyperkeratosis persisted, and skin appendages were slightly reduced compared to the EE group, indicating limited histological improvement.

Measurements of epidermal thickness presented in Figure 5B reveal that, in the IMQ-treated group, the epidermis thickened significantly compared to the control groups (103.33 ± 4.77). Unlike the groups treated with EE balm and CLO, in which thickness decreased significantly (51.67 ± 2.78 and 63.20 ± 3.27, respectively).

### Biochemical analyses

Table 1 reports serum lipid and biochemical data values for all groups. Total cholesterol differed significantly (p = 0.003) across the groups. IMQ administration led to an increase in total cholesterol compared with healthy controls, whereas treatment with EE balm reduced its levels. There was no significant difference in HDL-C between the groups (p = 0.262), while LDL-C levels were similar across all experimental groups (p = 0.242). In contrast, TG levels varied considerably between groups (p < 0.001); rats treated with IMQ, EE, and particularly clobetasol showed elevated levels compared to healthy controls.

**TABLE 1:** Evaluation of various biochemical parameters related to metabolic, hepatic, and renal function in all experimental groups.

Parameters	G I (negative control) (n = 6)	G II (IMQ) (n = 6)	G III (EE) (n = 6)	G IV(CLO) (n = 6)	p-value
TC (g/l)	0.60 ± 0,08^ab^	0.67 ± 0.09^a^	0.47 ± 0.08^b^	0.63 ± 0.03^a^	0.003*
HDL-C (g/l)	0.42 ± 0.02^a^	0.46 ± 0.06^a^	0.37 ± 0.04^a^	0.59 ± 0.17^a^	0.262
LDL-C (g/l)	0.13 ± 0.03^a^	0.15 ± 0.04^a^	0.12 ± 0.02^a^	0.12 ± 0.02^a^	0.242
TG (g/l)	0.47 ± 0.01^b^	0.56 ± 0.07^b^	0.61 ± 0.03^b^	0.83 ± 0.08^a^	<0.001 *
AST (U/I)	118.60 ± 31.22^a^	147.20 ± 38.75^a^	105.40 ± 50.68^a^	98.84 ± 5.38^a^	0.186
ALT (U/I)	39.20 ± 3.19^b^	52.80 ± 5.80^b^	62.20 ± 30.09^b^	89 ± 6.08^a^	0.001*
Urea (g/l)	0.46 ± 0.01^a^	0.51 ± 0.07^a^	0.49 ± 0.04^a^	0.50 ± 0.06^a^	0.517
Creatinine (mg/l)	4.61 ± 0.44^b^	4.64 ± 0.43^b^	6.03 ± 0.23^a^	5.06 ± 0.47^b^	<0.001 *
Total serum protein (mg/ml)	1.69 ± 0.08^a^	1.82 ± 0.013^a^	1.71 ± 0.08^a^	1.71 ± 0.21^a^	0.474
Total erythrocyte protein (mg/ml)	15.74 ± 1.65^a^	17.42 ± 1.15^a^	15.66 ± 1.12^a^	13.61 ± 2.27^a^	0.134

Significant differences (p < 0.05) between groups are shown in the data as mean ± standard deviation (SD).

AST activity was comparable across groups (p = 0.186), except for rats treated with IMQ, which showed slightly higher levels. ALT activity showed significant differences between groups (p = 0.001), with highest levels observed in animals treated with clobetasol. Rats treated with EE had moderate ALT activity, while the control and IMQ groups showed lower values. Urea concentrations remained stable across all groups (p = 0.517), while creatinine values varied significantly (p < 0.001), with the EE-treated group exhibiting the highest concentrations compared to the other groups. Overall serum protein concentrations remained consistent across all groups (p = 0.474), as did erythrocyte protein levels (p = 0.134).

Serum and erythrocyte oxidative stress parameters.

Oxidative stress parameters and antioxidant activities are presented in Table 2. Rats treated with 5% IMQ showed an elevated serum TBARS levels compared to healthy controls, reflecting increased lipid peroxidation. Both EE balm and clobetasol treatments markedly attenuated TBARS levels, indicating a reduction in oxidative damage (p < 0.001).

**TABLE 2:** Assessment of oxidative stress markers and antioxidant defenses at serum level in experimental groups.

Parameters	G I (negative control) (n = 6)	G II (IMQ) (n = 6)	G III (EE) (n = 6)	G IV (CLO) (n = 6)	p-value
TBARS (nmol/mg of protein)	2.47 ± 0.05^a^	7.55 ± 2.30^b^	2.26 ± 0.62^a^	2.97 ± 0.73^a^	<0.001 *
CAT (µmol/min/mg of protein)	5.30 ± 0.67^a^	3.06 ± 0.79^b^	4.24 ± 0.73^ab^	4.22 ± 0.86^ab^	0.003*
SOD (inhibition %/mg of protein)	15.21 ± 1.44^a^	03.17 ± 1.01^b^	18.24 ± 1.90^a^	17.27 ± 2.99^a^	0.000*
GPx (µmol/min/mg of protein)	28.46 ± 1.70^a^	18.52 ± 4.40^b^	31.25 ± 4.99^a^	29.70 ± 5.09^a^	0.001*
GST (µmol/min/mg of protein)	09.20 ± 2.15^a^	04.56 ± 1.76^b^	09.96 ± 2.29^a^	06.49 ± 0.93^a^	0.001*
GSH (µmol/mg of protein)	0.0047 ± 0.0009^a^	0.0021 ± 0.0007^b^	0.0042 ± 0.0011^a^	0.0033 ± 0.0007^a^	0.002*
Uric acid (mg/l)	22.47 ± 3.27^a^	43.46 ± 13.64^b^	09.72 ± 1.42^a^	16.38 ± 4.72^a^	<0.001 *

Significant differences (p < 0.05) between groups are shown in the data as mean ± standard deviation (SD).

CAT activity was suppressed in IMQ-treated animals compared to controls, whereas treatment with EE and clobetasol tended to restore its activity to baseline levels (p = 0.003). SOD and GPx activities decreased after IMQ exposure but were significantly elevated in the control, EE balm group, and clobétasol group (p < 0.001 and p = 0.001, respectively). GST activity decreased in IMQ-treated rats and was significantly restored by treatment with EE balm, while clobetasol resulted in a slight, non-significant improvement (p = 0.001).

GSH levels were lower in the IMQ group than in the control group and in animals treated with EE (p = 0.002). Serum uric acid concentrations were elevated after IMQ treatment, but they had decreased significantly in rats treated with EE balm and clobetasol compared to the IMQ group (p < 0.001).

Erythrocyte oxidative stress markers are reported in Table 3. IMQ treatment led to elevated TBARS levels relative to the control group, whereas rats treated with EE balm or clobetasol exhibited a marked reduction, indicating attenuation of lipid peroxidation (p = 0.005). IMQ group exhibited elevated activity of CAT, relative to all other groups., while both EE balm and clobetasol treatments brought it closer to baseline (p < 0.001). SOD activity decreased following IMQ exposure but showed partial recovery in the EE balm and clobetasol groups (p = 0.025). GPx activity was elevated in IMQ-treated rats; EE balm restored GPx to control values, whereas clobetasol treatment produced levels similar to the IMQ group (p = 0.001). GST activity was reduced in IMQ-treated animals but improved in the EE balm and control groups (p = 0.003). GSH content was lower in the IMQ group and increased following EE balm treatment, while clobetasol had a minimal effect (p = 0.004).

**TABLE 3:** Assessment of markers of oxidative stress and antioxidant defenses at erythrocyte level in the different experimental groups.

Parameters	Gi (negative control) (n = 6)	G II (IMQ) (n = 6)	Giii (EE) (n = 6)	G IV (CLO) (n = 6)	p-value
TBARS (nmol/mg of protein)	0.75 ± 0.40^a^	2.75 ± 0.66^b^	1.41 ± 0.24^a^	1.68 ± 1.09^ab^	0.005*
CAT (µmol/min/mg of protein)	04.26 ± 1.18^a^	13.85 ± 1.72^b^	05.25 ± 1.18^a^	06.11 ± 2.70^a^	<0.001 *
SOD (inhibition %/mg of protein)	258.64 ± 70.15^a^	135,52 ± 43.28^b^	225.17 ± 50.62^ab^	203.03 ± 61.96^ab^	0.025*
GPX (µmol/min/mg of protein)	24.56 ± 3.90^a^	50.21 ± 10.52^b^	32.84 ± 7.34^a^	39.04 ± 7.88^b^	0.001*
GST (µmol/min/mg of protein)	07.99 ± 2.09^a^	03.78 ± 1.27^b^	08.02 ± 2.10^a^	05.29 ± 1.21^ab^	0.003*
GSH (µmol/mg of protein)	0.0044 ± 0.0006^a^	0.0035 ± 0.0005^b^	0.0044 ± 0.0006^ab^	0.0037 ± 0.0005^b^	0.004*

Significant differences (p < 0.05) between groups are shown in the data as mean ± standard deviation (SD).

### TNF-α immunoassay

**FIGURE 6:**
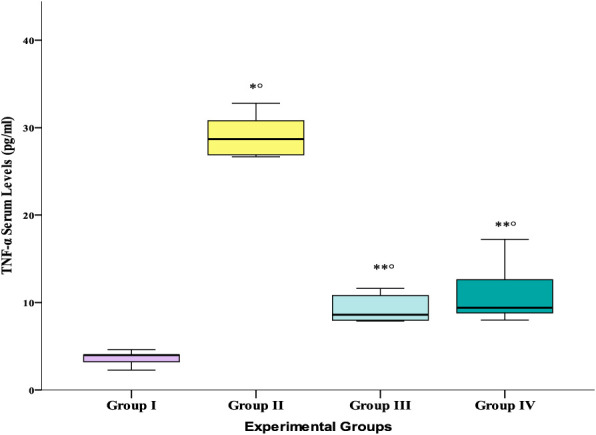
Serum TNF-α levels (pg/ml) across the different experimental groups. *° p < 0.01 compared to controls; **° p < 0.01 compared to the 5% IMQ group.

TNF-α levels were significantly elevated in IMQ group compared to healthy group. Administration of EE balm and 0.05% clobetasol significantly reduced TNF-α concentrations, indicating an effective attenuation of the inflammatory response (Figure 6).

## Discussion

Daily treatment with 5% IMQ for 10 days induced lesions with psoriatic characteristics in rats. The animals displayed progressive skin thickening, scaling, and marked erythema. Additionally, microscopic analysis showed a marked reduction in the granular layer and epidermal hyperplasia with parakeratosis and hyperkeratosis. These findings support earlier research and lend support to the IMQ model applicability in examining inflammatory pathways and treatment outcomes associated with psoriasis (Parmar et al., 2021; Shahine et al., 2023).

Clinical signs and the skin’s histological appearance both substantially improved with EE balm treatment. The epidermis regained a more typical structure by significant reduction in thickness suggesting a defensive response to the inflammation caused by IMQ. These findings could be attributed to the bioactive compounds of EE, such as cucurbitacins, flavonoids, phenolic acids, and fatty acids, as well as to the phenolic compounds in olive oil, including hydroxytyrosol, tyrosol, oleuropein, and ligstroside. As documented in several studies, the combined action of these compounds reduces the production of pro-inflammatory mediators (Bourebaba et al., 2020; Felhi et al., 2017; Khaleel et al., 2023; Pojero et al., 2023; Frumuzachi et al., 2024). A decrease in PASI scores supports the anti-inflammatory effect of EE observed in this model, which is consistent with previously published data (Sedlak and Lindsay, 1968). Notably, compared to 0.05% clobetasol propionate, EE balm demonstrated a similar ability to reduce psoriasiform lesions, highlighting the potential of plant metabolites as source of alternative therapeutic approaches in IMQ-induced psoriasis (Mejri et al., 2024).

Biochemical parameters revealed that animals treated with EE had lower total cholesterol and HDL levels than those in the clobetasol group, while LDL concentrations were similar. These changes may be linked to the phenolic compounds, flavonoids, and phytosterols present in *Ecballium elaterium*, which can influence lipid metabolism (Bourebaba et al., 2020). Cucurbitacins in particular, have been linked to several metabolic and cytotoxic effects that may influence circulating biochemical parameters. Consistent with previous findings reported by Anzano et al. (2024), these results suggest that EE may exert systemic metabolic effects following topical administration.

Furthermore, clobetasol treatment was associated with increased triglyceride and ALT levels, which may reflect a systemic topical toxicity (Kimura et al., 1986; Duong et al., 2025). Findings also support previous studies showing that prolonged exposure to topical corticosteroids leads to elevated liver enzymes and hepatocellular stress (Anderson and Borlak, 2008). Evaluation of renal function revealed that creatinine and urea levels remained within normal limits in control animals and those treated with clobetasol, while rats treated with EE showed significant elevation in creatinine unlike urea levels. This finding may reflects a temporary functional response or an early sign of renal toxicity due to systemic toxicity associated cucurbitacins, as reported in previous studies (Anzano et al., 2024). Total protein levels remained generally maintained in all experimental groups, with minor alterations that might be associated with metabolic changes associated with inflammation, as the latter has been linked to metabolic and antioxidant changes (Yin et al., 2013).

IMQ treatment induced a marked oxidative imbalance, as shown by increased TBARS levels in serum and erythrocytes, indicating increased lipid peroxidation. At the same time, antioxidant defenses were altered, with reduced SOD and CAT activities and changes in parameters related to GSH, GPx, and GST. The results of this study are in line with those of previous studies conducted in patients with psoriasis, which reported elevated TBARS levels associated with decreased SOD and CAT activities (Nemati et al., 2014; Kadam et al., 2010). Reduced erythrocyte SOD activity (Yildirim et al., 2003), decreased serum SOD levels (Megna et al., 2026), and lower erythrocyte GSH concentrations (Woźniak et al., 2007) have also been reported. In contrast, increased erythrocyte GPx activity has been reported in some studies (Skutnik-Radziszewska et al., 2020), which may reflect a compensatory response to persistent oxidative stress. Treatment with EE balm mitigated oxidative damage, as shown by decreased TBARS levels and partial to complete restoration of key antioxidant parameters, including SOD, GPx, GST, and GSH, depending on the tissue examined. These protective effects likely result from the combined antioxidant activity of *Ecballium elaterium* phytochemicals and the olive oil used as the formulation vehicle (Felhi et al., 2017).

IMQ treatment led to a significant increase in TNF-α, which was effectively reduced by both EE balm and clobetasol. Many studies have indeed indicated that the increase in TNF-α among IMQ-treated group may be amplified by accidental ingestion during grooming (Grine et al., 2016). Overall, these results align with previous reports showing that *Ecballium elaterium* can modulate key pro-inflammatory cytokines, including TNF-α and IL-6, which may underlie its anti-inflammatory activity (Arslan et al., 2016; Arslan et al., 2017).

Further studies involving vehicle control and active control groups are necessay to clarify the systemic safety profile of the balm EE. Such studies should include a phytochemical characterization of the balm, histopathological test and mechanistic analyses in order to determine if the observed increase in creatinine and ALT are reversible, associated with structural organ damage or due to the toxicity associated with bioactive compounds of *Ecballium elaterium* particularly cucurbitacins. Furthermore, studies should expand the cytokine panel (IL-17A, IL-22 and IL-23) to better elucidate the immunomodulatory mechanisms.

## Conclusion

This study shows that topical application of *Ecballium elaterium* balm significantly improved clinical signs, histological features, oxidative stress markers, and inflammatory responses caused by imiquimod in rats. These effects were partly associated with reduced oxidative stress and downregulation of TNF-α. Across several evaluated parameters, EE balm demonstrated efficacy comparable to clobetasol propionate, suggesting its potential as a plant-derived therapy for psoriasis. However, additional preclinical studies and carefully conducted clinical trials are required to verify its safety, and therapeutic efficacy.

## Data Availability

The original contributions presented in the study are included in the article/supplementary material, further inquiries can be directed to the corresponding authors.
